# Influence of daily imaging on plan quality and normal tissue toxicity for prostate cancer radiotherapy

**DOI:** 10.1186/s13014-016-0757-9

**Published:** 2017-01-10

**Authors:** Katharina Bell, Marina Heitfeld, Norbert Licht, Christian Rübe, Yvonne Dzierma

**Affiliations:** Department of Radiotherapy and Radiation Oncology, Saarland University Medical Centre, Kirrberger Str. Geb. 6.5, D-66421 Homburg/Saar, Germany

**Keywords:** Imaging dose distribution, Linac-based imaging, IGRT, NTCP modelling, Kilovoltage imaging, Megavoltage imaging, Prostate

## Abstract

**Background:**

Modern radiotherapy offers various possibilities for image guided verification of patient positioning. Different clinically relevant IGRT (image guided radiotherapy) scenarios were considered with regard to their influence on dosimetric plan quality and normal tissue complication probability (NTCP).

**Methods:**

This study is based on treatment plans of 50 prostate patients. We evaluate the clinically performed IGRT and simulate the influence of different daily IGRT scenarios on plan quality. Imaging doses of planar and cone-beam-CT (CBCT) images for three different energies (6 MV, 1 MV and 121 kV) were added to the treatment plans. The plan quality of the different scenarios was assessed by a visual inspection of the dose distribution and dose-volume-histogram (DVH) and a statistical analysis of DVH criteria. In addition, an assessment of the normal tissue complication probability was performed.

**Results:**

Daily 1MV-CBCTs result in undesirable high dose regions in the target volume. The DVH shows that the scenarios with actual imaging performed, daily kV-CBCT and daily 6MV imaging (1x CBCT, 4x planar images per week) do not differ exceedingly from the original plan; especially imaging with daily kV-CBCT has little influence to the sparing of organs at risk. In contrast, daily 1MV- CBCT entails an additional dose of up to two fraction doses. Due to the additional dose amount some DVH constraints for plan acceptability could no longer be satisfied, especially for the daily 1MV-CBCT scenario. This scenario also shows increased NTCP for the rectum.

**Conclusion:**

Daily kV-CBCT has negligible influence on plan quality and is commendable for the clinical routine. If no kV-modality is available, a daily IGRT scenario with one CBCT per week and planar axial images on the other days should be preferred over daily MV-CBCT.

## Background

Modern radiotherapy achieves highly conformal dose distributions even for complex-shaped target volumes, combining tumour coverage and sparing of organs at risk (OAR). The more precisely patient set-up can be achieved and maintained between and within treatment sessions, the more can safety margins be reduced and OAR avoided [1–4]. To optimally achieve this, image-guidance (IGRT) is a prerequisite, and it has been advocated that daily imaging should be performed. However, since most imaging techniques rely on ionizing radiation, each verification image entails additional dose to the patient.

The aim of this study is to assess in how far the plan quality and the modelled NTCP endpoints considered in treatment planning are affected by daily image-guidance for one of the most frequent IGRT indications, prostate cancer. Prostate radiotherapy is one of the main candidates for daily imaging due to inter-and intra-fraction mobility of the prostate and different filling levels of the surrounding organs (bladder and rectum). At the same time, the vicinity of these organs to the target volume means that very high dose gradients are involved, so that exact positioning must be verified [5–8].

A great number of different techniques are available for pre-treatment patient set-up verification, most relying on ionizing radiation, but differing in several respects: linac-based vs. external, planar vs. 3D (cone-beam CT), photon energy (kV or MV or some kind of intermediate energy photon beam). Furthermore, there is yet no consensus on whether daily imaging should be preferred over frequent, but non-daily verification. We therefore considered a set of realistic and representative imaging scenarios which are relevant to the clinical routine: Firstly, the original plan without imaging dose, which is the “gold standard” that was accepted for treatment (scenario 1). Moreover, the realistic imaging scenario carried out at our institution was included in the analysis as an example of a realistic clinical setting, although this did not involve daily, but 2–3 weekly imaging (scenario 2). On this basis, three scenarios for daily IGRT were simulated: a) daily kV-CBCT (cone-beam computed tomography) (scenario 3), which is the preferred option since image quality is best, while at the same time image dose is low [9]; b) daily IBL (image beam line) CBCT (scenario 4). The IBL has been marketed under the name kView, suggesting a kV-like energy spectrum, has a nominal energy of 1 MV and a carbon target [10, 11]. Therefore, users who are not equipped with “real” kV techniques may be tempted to use the IBL instead in a similar manner. c) In clinics where only the treatment beam (TBL) line is available for imaging, it is well-known that the additional imaging dose adds up considerably if daily CBCT are taken. We therefore opted for a scenario with one weekly 6 MV-CBCT and planar axial images on the other days (scenario 5).

Most studies so far have focussed on imaging dose by itself and not the clinical consequences on plan quality [12–17]. This is mainly indebted to the fact that imaging energies are not usually commissioned in the treatment planning system (TPS) and not included in the treatment plan. A few studies have calculated the imaging dose for each patient on the planning CT [17–21], but a systematic evaluation of different imaging scenarios on treatment plan quality is still missing. Furthermore, biological effects have been generally disregarded. We hence focussed on two aspects: firstly the dosimetric plan quality of the summation plan (treatment plus image guidance), including the dose distribution, dose-volume histogram (DVH), and planning objectives, and secondly the influence on normal-tissue complication probability (NTCP). For NTCP we considered biological endpoints for the OARs: rectum, bladder and femoral heads, in addition, regarding the rectum we also analysed clinically relevant endpoints like rectal bleeding and proctitis. To our knowledge, no such investigation is available in the literature.

## Methods

### Patient collective and treatment

This study is based on treatment plans of 50 prostate cancer patients retrospectively selected for a previous study [22], which analysed pre-treatment set-up images to determine set-up deviations. For the present study, the data from the previous study were used, with no further interaction with the patients. Therefore, an approval by the local ethics committee was not necessary due to the retrospective nature of this evaluation, however an approval of the institutional review board was obtained.

All patients received radiotherapy for two series. The planning target volume (PTV) encompassed the prostate (or the prostate bed after surgery), the seminal vesicles and the surrounding tissue of the small pelvis. For the shrinking field this was reduced in order to avoid high doses in the rectum. A total dose of 75 Gy was prescribed (1.8-2 Gy daily). All patients gave written informed consent for IGRT.

Our department is equipped with three Siemens (Siemens Healthcare, Erlangen, Germany) linear accelerators, two Artistes and one Oncor, with matched energies. All of them can be operated in the 6MV photon mode, one Artiste disposes of the flattening filter free 7MV photon energy, the other Artiste and the Oncor can additionally be operated in the 18MV photon mode.

Treatment planning was carried out on the basis of a planning CT acquired with a Philips Brilliance BigBore 120 kV (Philips Healthcare, DA Best, Netherlands). Depending on the patient anatomy and resulting target coverage and dose to OAR, either a 3D-conformal technique with 18 MV photon beams or (more frequently) IMRT with 6 MV was applied for the PTV. The shrinking field was always planned as 6 MV IMRT. Planning was performed in the Philips Pinnacle TPS V9.2 (Philips Healthcare, DA Best, Netherlands), using the collapsed cone algorithm and a 2 mm dose grid.

### Imaging scenarios and dose calculation

All three imaging techniques considered here are available at our institution: a TBL with 6 MV, the IBL of nominally 1 MV and a kV system using 70–140 kV, which uses 121 kV for prostate imaging (both axial planar images and kV-CBCT). The kV-system is available only at one of our Artistes, while both Artistes are equipped with the IBL modality. TBL imaging is feasible with all three linear accelerators. All three energies were dosimetrically characterized and are commissioned in the TPS, so that imaging dose distributions could be calculated for each patient. The modelling of the image beam line was performed using the Pinnacle automatic modelling routine presenting a very stable inversion in relation to a starting spectrum selection [23]. kV-CBCT modelling in the TPS required the addition of photon energy deposition kernels for low photon energies. After their inclusion, modelling the percent-depth-dose curve and beam profiles could be carried out similarly to standard commissioning of the treatment beam [24].

The imaging scenarios are listed in Table 1. The original plan does not include any imaging dose. Although it would be desirable to include the imaging dose at the time of treatment planning, this is usually not the case in the clinical routine, so that we take this plan as the “gold standard” on which the decision to accept a plan for treatment is normally based.

**Table 1:** Imaging scenarios

Scenario 1	Scenario 2	Scenario 3	Scenario 4	Scenario 5
Original plan	Actual imaging performed (non-daily)	Daily kV CBCT	Daily IBL CBCT	Daily imaging: 1xTBL CBCT, 4xTBL planar images per week

Scenario 2 is the realistic case that was carried out at our institution. As our department has three linear accelerators with matched energies and identical 160 MLC, the choice where the patients should be treated is based on the IGRT technique available at the machines. Only one linac offers kV imaging, two IBL, and all the TBL. In principle, prostate patients are preferentially assigned to the machine with kV capability due to the better soft-tissue contrast. However, due to maintenance or repair, sometimes also the clinical schedule, patients are occasionally shifted between the machines, so that different combinations of kV, IBL and TBL images are taken. Images were taken approximately every other fraction, and about every third image was a CBCT. A detailed description of the imaging scenario can be found in [22].

Scenario 3 considers daily kV CBCT with 360°. The kVision system uses an auto-exposure technique based on a pre-shot, so that mAs values depend on the patient diameter. For the patient collective, mAs values ranged between 439 mAs and 1548 mAs per kV-CBCT. For each patient, the average mAs of all kV-CBCTs was used in the simulation of daily kV-CBCT. For those patients who did not receive kV-CBCT, the simulation used the average mAs of all patients.

IBL-CBCT and TBL-CBCT as simulated in scenarios 4 and 5 were taken with a full gantry rotation and a user-defined monitor setting of between 7 and 16 monitor units (MU) depending on patient size. Similarly to scenario 3, for each patient the average MU from the realistic imaging performed were taken for the simulation; in cases where this was not available the average over all patient MU settings was applied. For the TBL-CBCTs the overall mean value was 16 MU, for IBL-CBCT 15 MU and for kV-CBCT 779 mAs. Planar axial images were taken with gantry angles of 0° and 90° with 1 MU each.

### Dosimetric plan quality evaluation

The plan quality of the different scenarios was assessed by evaluating the dose distributions and different dose-volume objectives. Table 4 lists DVH criteria applied during planning and in plan acceptance.

In addition to a visual inspection of the dose distribution a statistical analysis was performed using a one-way ANOVA with repeated measures. The different IGRT scenarios were compaired pairwise with the Wilcoxon-signed-rank test. Moreover, it was assessed how many times the plans with IGRT failed the acceptance criteria that were passed by the original plan (Scenario 1).

### Normal tissue complication probability

For biological endpoints of the relevant OARs we used the NTCP model implemented in the Pinnacle TPS (Table 2). The formalism is based on the Källmann S-model [25, 26]:

**Table 2:** Parameter and endpoints for NTCP calculation

ROI	D_50_	γ	α/β	s	Endpoint
Bladder	80 Gy	3.0	3 Gy	0.18	Symptomatical contracture
Rectum	80 Gy	2.2	3 Gy	1.50	Necrosis/Stenosis
Posterior rectal wall	80 Gy	2.2	3 Gy	1.50	Necrosis/Stenosis
Femoral heads	65 Gy	2.7	3 Gy	1.00	Necrosis

$$ {NTCP}_j={\left[1-{\displaystyle {\prod}_{i=1}^M{\left[1-{P_{ij}}^{s_j}\right]}^{\varDelta {v}_{ij}}}\right]}^{1/{s}_j} $$

with

*NTCP*_*j*_: probability of causing normal tissue complication for organ *j*

*M*: number of voxels in the organ *j*

*P*_*ij*_: Tumor control probability in voxel *i* of organ *j*

*s*_*j*_: relative seriality of the organ *j*

*Δv*_*ij*_: relative volume of a voxel *i* of the organ *j*

We additionally considered some relevant clinical rectal toxicity endpoints (rectal bleeding grades 1 and 2, proctitis grades 1 and 2, rectal bleeding grade 2 only and proctitis grade 2 only). As parameters were only available for the Lyman Kutcher Burman (LKB) model for these endpoints, they were calculated in this model using parameter values from Gulliford et al. [27] (Table 3). With the LKB model NTCP for non-uniformly irradiated volumes is calculated using the histogram reduction method and the following equation:

**Table 3:** Parameters for the LKB model for rectal toxicities [27]

Enpoint (toxicity grades)	TD50 (Gy)	Slope m	Volume factor n
Rectal bleeding (G1&2)	59.2	0.29	0.17
Proctitis (G1&2)	57.3	0.33	0.2
Rectal bleeding (G2)	68.9	0.16	0.18
Proctitis (G2)	68.3	0.22	0.17

$$ NTCP(volume)=0.5+\frac{\mathrm{erf}\left(\frac{t}{\sqrt{2}}\right)}{2.0} $$

where

$$ t=\frac{Dose-TD50(volume)}{m\cdot TD50(volume)} $$

$$ \mathrm{T}\mathrm{D}50\left(\mathrm{volume}\right)=\mathrm{T}\mathrm{D}50\cdot {\mathrm{volume}}^{{\textstyle \hbox{-}}\mathrm{n}} $$

n is the volume factor for the structure, TD50 is the dose at 50% probability of complication for the structure.

m is the slope factor:

$$ m=\frac{1}{\sqrt{2\pi} \cdot {\gamma}_{50}} $$

Dose is the maximum dose to the structure, volume is the effective volume computed from the dose volume histogram [28].

## Results

### Dosimetric evaluation of the plan quality

**Fig. 1:**
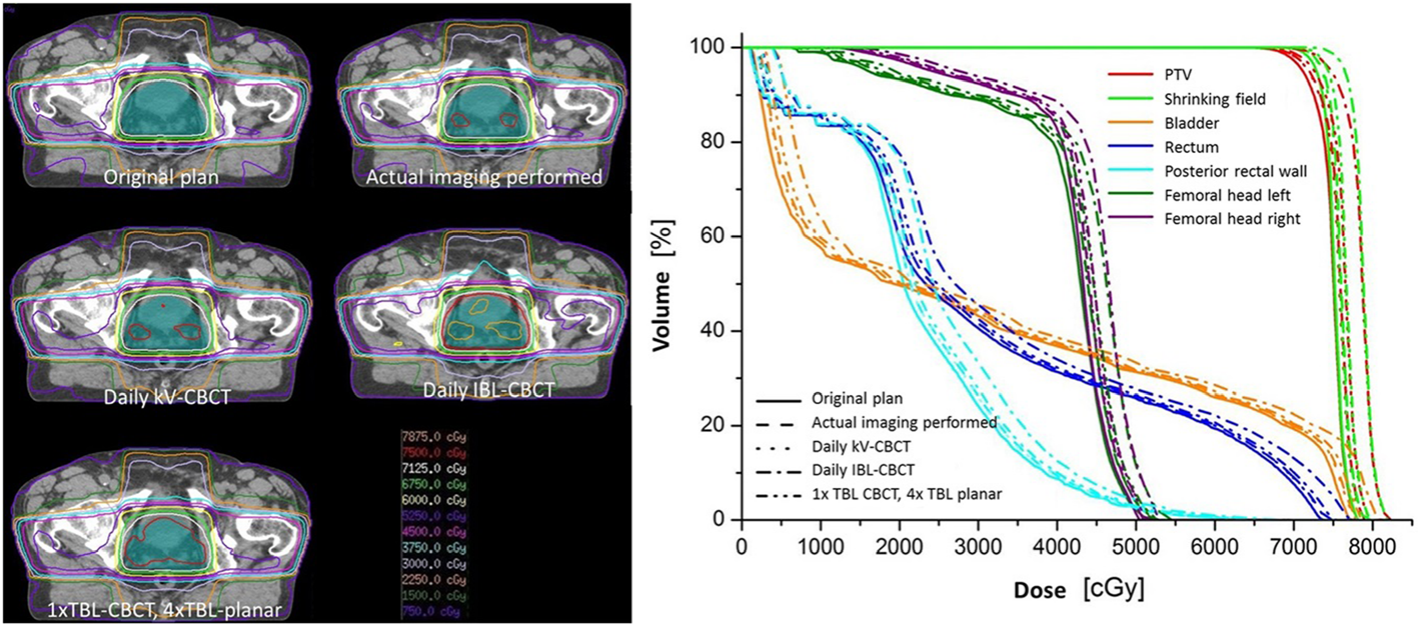
Example dose distribution and DVH of one patient for all scenarios

An example of the dose distributions for all scenarios is shown in Fig. 1. Obviously, the additional imaging dose does not compromise PTV coverage. However, the use of a daily IBL-CBCT causes some undesirable high dose regions. Although slight changes in the lower dose regions can be discerned, these can be better interpreted based on the DVH and plan acceptability criteria.

Figure 1 also shows one example DVH including all scenarios. While the original plan without imaging naturally has lowest dose, scenario 2, 3 and 5 do not differ exceedingly from the original plan. Especially the real IGRT and the daily kV-CBCT do not have great influence on the OAR. Contrarily, daily IBL-CBCT causes a pronounced shift in the DVH with an additional dose of up to two fraction doses (on occasions, over 4 Gy).

Due to the concomitant imaging dose the volumetric percentage of the OAR receiving a particular dose increases. Accordingly, some DVH constraints for plan acceptability may no longer be satisfied. Table 4 lists the number of patients for which a given DVH objective was exceeded for the different scenarios (but satisfied by the original plan).

**Table 4:** DVH criteria for plan acceptability and number of patients with exceeded DVH objectives for the different scenarios

Organ	Criteria	Scenario 2	Scenario 3	Scenario 4	Scenario 5
Bladder	V75Gy < 15%	0	0	10	3
	V70Gy < 20%	1	3	7	4
	V65Gy < 50%	2	2	3	2
	V50Gy < 50%	0	0	2	1
Rectum	V70Gy < 10%	0	2	14	9
	V60Gy < 30%	0	0	7	2
	V50Gy < 50%	0	0	0	0
	V40Gy < 70%	0	0	1	0
	V30Gy < 80%	1	1	2	1
Posterior rectal wall	V50Gy < 15%	0	0	3	0
	V40Gy < 30%	2	1	5	3
	D2% < 60Gy	0	0	4	1
Femoral heads	V50Gy < 5% le	1	2	7	4
	V50Gy < 5% ri	2	2	9	3

Nearly all DVH criteria passed by the original plan were equally satisfied by the realistic IGRT and the daily kV-CBCT scenarios. Only in few cases (0–3) were the criteria no longer met after imaging dose was included. Plan acceptability was hence not markedly compromised by imaging.

In contrast, daily IBL-CBCT caused the DVH criteria to be exceeded in a notable number of cases (up to 14 for the rectum V70Gy). Almost every DVH objective was affected by this, some frequently.

Despite the even higher MV photon energy, the TBL scenario improved plan acceptability in comparison with daily IBL-CBCT because of the dose reduction by replacing four 6 MV-CBCT by axial images. Some DVH constraints were always or nearly always met even with scenario 5 (rectum V50Gy, V40Gy, posterior rectal wall V50 Gy, also bladder V50 Gy, V65 Gy, etc.). Rectum V70Gy < 10% was exceeded for 9 patients; this was the only objective to be exceeded for more than 4 patients.

Mean DVH metrics are given in Table 5. These values confirm the findings above, with scenario 2 causing the smallest effects on the plan quality, followed by scenario 3. Daily imaging with IBL-CBCT lead to a noticeable increase in OAR dose, reflected in an increase (up to 5%) in the volume of OAR receiving a given dose. The effects of the 6 MV scenario were larger than scenarios 2 and 3, but considerably smaller than scenario 4.

**Table 5:** Mean values ± standard deviations and lower - upper 95% confidence levels of the DVH criteria for the different scenarios

Organ	DHV Metric (Acceptance Criteria)	Scenario 1	Scenario 2	Scenario 3	Scenario 4	Scenario 5
Bladder [%]	V75Gy (< 15%)	2.85 ± 4.44 (1.59-4.11)	3.88 ± 5.46 (2.33-5.44)	4.28 ± 6.04 (2.56-5.99)	7.81 ± 10.4 (4.87-10.76)	5.73 ± 7.82 (3.50-7.95)
	V70Gy (< 20%)	9.19 ± 11.3 (5.97-12.41)	9.53 ± 11.6 (6.23-12.83)	9.64 ± 11.7 (6.31-12.98)	14.3 ± 12.7 (10.70-17.90)	10.2 ± 12.1 (6.75-13.61)
	V65Gy (< 50%)	20.6 ± 13.6 (16.70-24.41)	21.9 ± 14.2 (17.84-25.91)	21.9 ± 14.1 (17.86-25.88)	25.4 ± 15.2 (21.08-29.69)	23.1 ± 14.2 (19.01-27.10)
	V50Gy (< 50%)	34.0 ± 17.9 (28.87-39.07)	34.4 ± 18.0 (29.23-39.49)	34.4 ± 18.1 (29.29-39.55)	36.1 ± 18.6 (30.84-41.42)	34.9 ± 18.2 (29.72-40.05)
Rectum [%]	V70Gy (< 10%)	4.63 ± 4.98 (3.22-6.05)	5.1 ± 5.39 (3.57-6.63)	5.28 ± 5.58 (3.70-6.87)	7.83 ± 7.36 (5.74-9.92)	5.89 ± 6.12 (4.15-7.63)
	V60Gy (< 30%)	18.4 ± 6.60 (16.55-20.31)	19.2 ± 6.60 (17.34-21.09)	19.4 ± 6.76 (17.43-21.27)	22.6 ± 7.07 (20.62-24.64)	20.2 ± 6.78 (18.32-22.17)
	V50Gy (< 50%)	30.5 ± 8.81 (28.00-33.01)	31.1 ± 8.91 (28.59-33.66)	31.3 ± 9.07 (28.67-33.83)	34.0 ± 9.73 (31.20-36.73)	31.9 ± 9.22 (29.31-34.55)
	V40Gy (< 70%)	41.4 ± 12.0 (37.97-44.80)	42.1 ± 12.3 (38.61-45.60)	42.2 ± 12.4 (38.72-45.75)	45.6 ± 13.7 (41.73-49.49)	43.1 ± 12.6 (39.46-46.65)
	V30Gy (< 80%)	56.4 ± 17.0 (51.60-61.24)	56.3 ± 17.1 (51.45-61.17)	56.6 ± 17.2 (51.72-61.50)	60.8 ± 17.7 (55.80-65.87)	57.6 ± 17.3 (52.69-62.54)
Posterior rectal wall [%]	V50Gy (< 15%)	2.84 ± 3.01 (1.99-3.69)	3.10 ± 3.15 (2.21-4.00)	3.23 ± 3.32 (2.29-4.18)	4.94 ± 4.61 (3.63-6.25)	3.60 ± 3.59 (2.58-4.62)
	V40Gy (< 30%)	12.2 ± 9.25 (9.59-14.85)	13.2 ± 9.92 (10.33-15.97)	13.3 ± 10.2 (10.43-16.22)	18.1 ± 13.7 (14.21-21.97)	14.4 ± 10.8 (11.30-17.44)
	D2% (< 60Gy) [Gy]	49.0 ± 7.23 (46.95-51.06)	49.6 ± 7.16 (47.56-51.63)	49.7 ± 7.25 (47.63-51.75)	52.3 ± 7.31 (50.25-54.41)	50.4 ± 7.25 (48.30-52.42)
Femoral heads [%]	V50Gy (< 5%) le	0.66 ± 1.70 (0,17-1,14)	0.88 ± 2.26 (0,24-1,52)	1.05 ± 2.71 (0,28-1,83)	3.32 ± 6.72 (1,42-5,23)	1.49 ± 3.94 (0,37-2,61)
	V50Gy (< 5%) ri	0.52 ± 1.26 (0,17-0,89)	0.76 ± 1.71 (0,28-1,25)	0.96 ± 2.12 (0,36-1,56)	3.61 ± 7.92 (1,36-5.86)	1.31 ± 3.02 (0,45-2,17)

The statistical analysis in most cases resulted in highly significant differences between the scenarios (for *p*-values see Tables 7 and 8), especially for the comparisons with the IBL-scenario. Only the real imaging scenario in comparison to the kV-scenario was not significant for a number of DVH constraints (V65Gy and V60Gy of the bladder and V60Gy of the rectum).

### Normal tissue complication probability

**Fig. 2:**
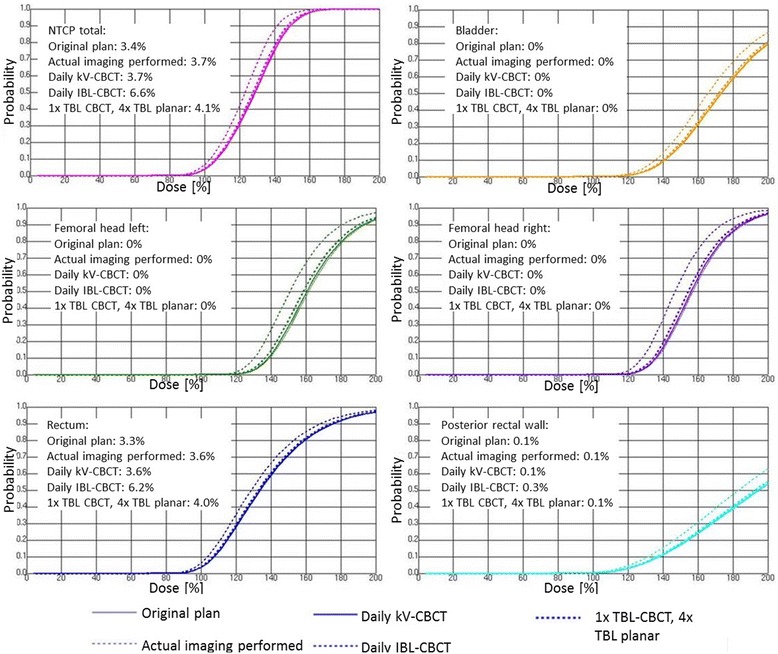
Dose–response curves for NTCP

The dose–response-curves for the NTCP show a typical sigmoidal course. For the biological endpoints (necrosis, stenosis, contracture – see Table 2) the NTCP for all OARs is small (Fig. 2), which was expected since the DVH objectives for planning (in addition to following the ALARA principle) are explicitly chosen in a way to avoid normal tissue toxicity. The only organ with more than 1% NTCP is the rectum, so we disregard the bladder and femoral heads and focus below on the clinically more relevant endpoints for rectal toxicity. Average values for all patients for the different scenarios are given in Table 6. Rectal bleeding and proctitis grades 1 and 2 show probabilities for toxicities up to 40%. Considering only grade 2 toxicities, NTCP values decrease down to 6–10% for rectal bleeding and to 14–20% for proctitis. The differences in NTCP between the scenarios 2, 3 and 5 are minor, with less than 3% increase. However, the IBL-CBCT scenario leads to increased NTCP by up to 6% in comparison with scenario 1.

**Table 6:** Mean values ± standard deviations and lower - upper 95% confidence levels of the NTCP-analysis for the different scenarios

Organ	Scenario 1	Scenario 2	Scenario 3	Scenario 4	Scenario 5
Necrosis [%]	2.24 ± 0.21 (1.81-2.67)	2.46 ± 0.21 (2.03-2.89)	2.52 ± 0.23 (2.06-2.98)	3.86 ± 0.32 (3.22-4.50)	2.88 ± 0.25 (2.37-3.38)
Rectal bleeding (G1&2) [%]	34.02 ± 1.02 (31.98-36.06)	35.08 ± 1.01 (33.04-37.12)	35.2 ± 1.04 (33.11-37.29)	39.98 ± 1.12 (37.74-42.22)	36.46 ± 1.05 (34.35-38.57)
Proctitis (G1&2) [%]	35.88 ± 0.98 (33.91-37.85)	36.88 ± 0.98 (34.91-38.85)	36.98 ± 1.00 (34.96-39.00)	41.34 ± 1.06 (39.21-43.47)	38.12 ± 1.02 (36.08-40.16)
Rectal bleeding (G2) [%]	6.48 ± 0.54 (5.40-7.56)	7.06 ± 0.55 (5.95-8.17)	7.2 ± 0.58 (6.06-8.37)	10.22 ± 0.78 (8.66-11.78)	7.86 ± 0.63 (6.60-9.12)
Proctitis (G2) [%]	14.5 ± 0.7 (13.09-15.91)	15.28 ± 0.73 (13.82-16.74)	15.3 ± 0.74 (13.81-16.79)	19.02 ± 0.89 (17.24-20.80)	16.22 ± 0.78 (14.65-17.79)

Again the statistical analysis shows highly significant differences between the scenarios, only scenario 2 vs. 3 is not significant for all NTCP endpoints (Tables 7 and 8).

**Table 7:** *p*-values of the statistical analysis, lower bound for the ANOVA and Wilk’s Lambda for the multivariate tests

Organ	Criteria	ANOVA –Lower-bound	Multivariate tests – Wilk’s Lambda
Bladder	V75Gy	<0.001	<0.001
	V70Gy	<0.001	<0.001
	V65Gy	<0.001	<0.001
	V50Gy	<0.001	<0.001
Rectum	V70Gy	<0.001	<0.001
	V60Gy	<0.001	<0.001
	V50Gy	<0.001	<0.001
	V40Gy	<0.001	<0.001
	V30Gy	<0.001	<0.001
Posterior rectal wall	V50Gy	<0.001	<0.001
	V40Gy	<0.001	<0.001
	D2%	<0.001	<0.001
Femoral heads	V50Gy le	<0.001	0.0040
	V50Gy ri	0.0035	0.0084
	Necrosis	<0.001	<0.001
NTCP	Rectal bleeding (G1&2)	<0.001	<0.001
	Proctitis (G1&2)	<0.001	<0.001
	Rectal bleeding (G2)	<0.001	<0.001
	Proctitis (G2)	<0.001	<0.001

**Table 8:** *p*-values of the pair-wise Wilcoxon signed rank tests

Organ	Criteria	Scenario 2 vs. 3	Scenario 2 vs. 4	Scenario 2 vs. 5	Scenario 3 vs. 4	Scenario 3 vs. 5	Scenario 4 vs. 5
Bladder	V75Gy	0.004	<0.001	<0.001	<0.001	<0.001	<0.001
	V70Gy	0.0065	<0.001	<0.001	<0.001	<0.001	<0.001
	V65Gy	n.s.	<0.001	<0.001	<0.001	<0.001	<0.001
	V50Gy	n.s.	<0.001	<0.001	<0.001	<0.001	<0.001
Rectum	V70Gy	0.0029	<0.001	<0.001	<0.001	<0.001	<0.001
	V60Gy	n.s.	<0.001	<0.001	<0.001	<0.001	<0.001
	V50Gy	0.020	<0.001	<0.001	<0.001	<0.001	<0.001
	V40Gy	0.021	<0.001	<0.001	<0.001	<0.001	<0.001
	V30Gy	0.003	<0.001	<0.001	<0.001	<0.001	<0.001
Posterior rectal wall	V50Gy	0.003	<0.001	<0.001	<0.001	<0.001	<0.001
	V40Gy	0.024	<0.001	<0.001	<0.001	<0.001	<0.001
	D2%	0.050	<0.001	<0.001	<0.001	<0.001	<0.001
Femoral heads	V50Gy le	0.021	<0.001	<0.001	<0.001	<0.001	<0.001
	V50Gy ri	0.010	<0.001	<0.001	<0.001	0.001	<0.001
	Necrosis	n.s.	<0.001	<0.001	<0.001	<0.001	<0.001
NTCP	Rectal bleeding (G1&2)	n.s.	<0.001	<0.001	<0.001	<0.001	<0.001
	Proctitis (G1&2)	n.s.	<0.001	<0.001	<0.001	<0.001	<0.001
	Rectal bleeding (G2)	n.s.	<0.001	<0.001	<0.001	<0.001	<0.001
	Proctitis (G2)	n.s.	<0.001	<0.001	<0.001	<0.001	<0.001

(*n.s.* not significant)

## Discussion

### Comparison with previous studies

Most of the current literature regarding imaging dose has focussed on the additional dose itself for different modalities, especially kV-imaging. Amer et al. [13] assessed additional doses of kV-CBCT from the Elekta Synergy X-Ray system. They measured imaging doses on various locations in a Rando phantom and at patients’surfaces. The weighted doses for their in-house CBCT protocol were 1.6 mGy for the head, 6 mGy for the lung and 22 mGy for the pelvis.

Similar results can be found in Ariyaratne et al. [15] with a nominal measured concomitant dose to the pelvis of 30 mGy per CBCT exposure and in Schneider et al. [16] who concluded that most imaging methods, including pelvis CBCT protocols add no more extra dose to the patient than the therapy dose variations between different treatment techniques (e.g., IMRT in contrast to 3D conformal treatment).

Dzierma et al. [17] evaluated the imaging dose and dose distribution for three different modalities. For the kV modality the findings fit well with the literature mentioned above (kV-CBCT resulted in a dose of about 25 mGy for the prostate and 3–9 mGy for head and neck cases). Moreover they showed for head and neck an extra dose of 80 mGy for 6 MV CBCT and 34–62 mGy for IBL CBCT. For the prostate the 6 MV CBCT result in an imaging dose of 120–150 mGy and 80–110 mGy for IBL CBCT.

The few other studies to be found that calculated the imaging dose on the planning CT did not systematically evaluate different IGRT scenarios but focussed on kV modalities [18–21]. Alaei et al. [20] showed that the dose from daily kV CBCT results in an additional isocenter dose of the order of 30–40 mGy for 35 fractions head and neck and 230–240 mGy for 25 fractions pelvis irradiation. The authors used the commissioning of kV CBCT beams in the TPS primarily to include the imaging dose in the treatment plan prior to optimization.

Overall, this study is the first to systematically analyse the influence of different imaging scenarios on treatment plan quality with respect to changing DVH constraints and biological/clinical endpoints.

### Discussion of normal tissue complication probability

The NTCP calculations were performed with the biological response panel implemented in the Pinnacle TPS using information of the Källmann S-model with appropriate endpoints for the relevant OARs (Table 2). As it is the aim of every treatment plan to avoid normal tissue toxicities, the endpoints like necrosis and stenosis show probabilities of about 0% for most cases and OARs – this is natural since the DVH constraints required for plan acceptability in our institution rely on the clinical information from the QUANTEC studies. Only the rectum showed NTCP values of more than 1%, which is why only this OAR was followed further. For the rectum, different clinically interesting endpoints were considered. The incidence of late gastrointestinal toxicity after prostate cancer treatment is a widely studied complication. Side effects like rectal bleeding or proctitis are no uncommon complications. To take these clinical toxicities into account many studies deal with the calculation of NTCP parameters for specific rectal complications [27, 29–31]. Gulliford et al. [27] estimated parameters for the Lyman-Kutcher-Burman model for different rectal complications of different grades observed in clinical practice. As rectal bleeding is the most frequently reported rectal toxicity, we assessed in our study the influence of the different IGRT scenarios on this toxicity, moreover we chose proctitis as a second important rectal complication. The Pinnacle TPS offers the possibility to obtain NTCP response values for a ROI using the Lyman-Kutcher-Burman model. With the parameters “Dose at 50% probability”, “slope factor” and “volume factor”, NTCP calculations for all organs and endpoints could be performed. Using the results of Gulliford et al. (Table 3) we estimated NTCP for the different imaging scenarios regarding rectal bleeding and proctitis grades 1 and 2 and rectal bleeding and proctitis grade 2 only to evaluate clinical consequences of the concomitant imaging dose. The use of two different NTCP models was necessary since the overall OAR complication parameters were only available for the Källmann-S-model, whereas the model parameters for the rectum clinical endpoints could only be found for the Lyman-Kutcher-Burman model.

### Practical implications of the results

We have considered different realistic and clinically relevant IGRT scenarios: Scenario 1 considers the original plan representing the planned and accepted case for treatment. This scenario provides a reference to evaluate the scenarios including imaging doses.

At our institution the frequency and technique of the setup images differ for each patient due to shifting between three machines with matched energies but different imaging modalities. Therefore, imaging dose is not a-priori included in the treatment plan. This is acceptable since extensive previous tests have shown that the realistic imaging dose accumulated shows only minimal effects on plan quality. This is demonstrated again in scenario 2, which reflects the actual IGRT received by each patient. While this involved non-daily imaging, it shows only minimal effects on the plan quality; the majority of plans remains within the acceptance criteria and the additional dose contribution is marginal. In this regard, it must be stressed that the “real” scenario (2) that represents the imaging performed at our institution entails higher positioning uncertainty than those scenarios with daily IGRT, so that dose deviations arising from positioning errors come into effect. Therefore, although this scenario involves only little additional imaging dose and hence a minor effect of IGRT dose on plan quality, the possible positioning errors themselves may compromise plan quality.

The trade of between imaging dose and setup errors is highly relevant and should be considered in future studies. In the present work, the setup accuracy for the three scenarios with daily imaging can be considered identical and optimal, so positioning errors can be neglected in the analysis for these plans and the three daily imaging scenarios can be directly compared based on the imaging dose. However, for the non-daily scenario (the realistic case), statistical setup errors can be expected to have remained present on the days without imaging, leading to some degradation of plan quality. Therefore, this plan cannot directly be compared with the daily imaging scenarios. Similarly, the original plan without imaging does certainly not correspond to the dose distribution that would be expected if no imaging was ever performed. Importantly, we do not mean to suggest this scenario (no imaging) as a possible alternative. Rather, this scenario should provide the baseline against which the scenarios including imaging should be compared. This should sensitize us to the fact that the usual way of accepting a plan for treatment – which is generally without any inclusion of imaging dose – might not show any infringement of DVH constraints that might be created by the additional imaging dose.

If *daily* IGRT is desired, a suitable scenario must be chosen depending on the available imaging techniques. For daily kV-CBCT, the additional dose contribution has only minor impact on plan quality and NTCP. Regarding the additional dose several studies and our own measurements have found skin doses for kV imaging the range of 1–3 cGy per image [32]. While this is not negligible, the influence on plan quality is very small and can be justified by the improvement in set-up accuracy. This scenario is hence most desirable, since it combines lowest concomitant dose with best image quality (soft-tissue contrast). This offers the possibility of performing image registration based on soft tissues rather than bony structures, so that motion of the prostate, bladder and rectum relative to the bony landmarks and relative to each other can be compensated for. Where this is available, this scenario can be integrated into the clinical routine without further difficulty.

However, since not every institution is equipped with a kV-modality, we considered two alternative scenarios. Daily IBL-CBCT results in a concomitant dose of up to two additional fraction doses. Due to this additional dose, the DVH constraints leading to plan acceptability are no longer met for many cases (70 deviations from the acceptance criteria of the original plan). Moreover, high dose regions are induced in the PTV. The NTCP is increased by up to 6% (proctitis grades 1 and 2: scenario 1: 35.88%, scenario 4: 41.34%). Although these are only model values, a possible clinical relevance of the change in dose should be kept in mind. We must stress that the IBL technique has been marketed in some cases as “kV-like”, which might leave the impression that it can be employed as frequently as a “real” kV modality. As our results demonstrate, this is not the case and the IBL, while improving image quality and dose when compared to 6 MV, is still an MV-photon beam and should be employed with discretion.

Finally, unavailability of a kV technique does not necessarily mean that daily IGRT must be abandoned. As shown for the TBL, a scenario with daily axial images and interspersed MV-CBCT can be designed so that plan quality is scarcely compromised. Depending on the frequency of CBCT, the planning criteria can be met in most cases, and the simulated effect on NTCP is minor. If IBL is available, a similar scenario could be adopted, with a further reduction in dose.

All scenarios relying on MV imaging (TBL or IBL) suffer from the draw-back that soft tissues can hardly be discerned, even in CBCT images. Therefore, image fusion and set-up corrections will primarily rely on bony structures. This will be incapable of showing real prostate motion within the body and different positions and size of bladder and rectum. Therefore, larger safety margins will need to be applied in PTV delineation and treatment planning. When this is kept in mind, however, daily imaging can be performed with these techniques. Furthermore, since this relies on bony structures, the information loss in applying axial planar imaging rather than CBCT is not as marked. Using exclusively kV, an image fusion on soft tissue would be possible leading to advantages in positioning precision. Moreover the use of gold markers would be a good solution for the prostate. However, as this work primarily deals with the additional imaging dose without taking into account potential setup errors, we assume that the imaging modalities are able to control and correct setup errors in the same way.

If daily volumetric imaging is desired in the absence of a kV modality, a possibility is to include the additional imaging dose in treatment planning. Including the planned imaging dose in the treatment plan would give a realistic approximation of the total dose and allow to optimize the treatment in such a way that PTV hot-spots and exceeding exposure of OARs can be avoided.

In this study, dose calculation accuracy for the MV beams is comparable to standard requirements for commissioned treatment beams; for the kV energy, the accuracy is reduced since the dose calculation algorithms are optimized for the energy range dominated by Compton scattering. For the low energy photons where the photo effect comes into play, deviations from the measurements at beam commissioning were mostly below 10%, but could reach up to 30% in the close vicinity to bony structures [24]. This might slightly affect the doses at the femoral heads, but should introduce only minor errors in the results for the bladder and rectum.

In addition to the aspects of imaging dose and positioning accuracy, the time required for imaging plays an important role in the clinical context. In principle, the additional time amount for the different imaging modalities is irrespective of the used energy, so that 6 MV and 1 MV imaging is identical from the point of view of imaging time. In both cases, the flat panel needs to be deployed opposite to the collimator (about 15 s). For kV imaging, both a flat panel and the X-ray tube must be rotated into the beam line (about 25 s). After deployment, performing volumetric imaging requires more time than recording two planar images. CBCTs with a full gantry rotation of 360° take about one minute and 30 s, while the two planar projections including gantry movement can be taken in less than one minute. However, the most time-consuming part in IGRT is the merging and adaption of the verification images with the images of the planning CT, in particular the selection of the planning CT images or digitally reconstructed radiographs from the database. After this, the auto-fusion algorithm runs in about 50 s for CBCTs. If manual intervention is required, the time for image fusion can be longer.

## Conclusion

As modern radiation therapy offers the possibility for steeper dose gradients leading to smaller safety margins, it is essential to perform setup verification images regularly. To evaluate the influence of different IGRT protocols on plan quality, several clinically relevant scenarios were simulated for a collective of 50 prostate cancer patients. Daily kV-CBCT has smallest influence on plan quality and is commendable for the clinical routine. If no kV-modality is available, daily MV-CBCT – even the nominal 1 MV image beam line – should not be used without keeping in mind and possibly adjusting for the additional imaging dose. A daily IGRT scenario with mostly planar axial images and intermittent CBCT might be preferred. For these recommended scenarios, the model results in less than 1% increase in NTCP.
